# Sexual modulation in a polyploid grass: a reproductive contest between environmentally inducible sexual and genetically dominant apomictic pathways

**DOI:** 10.1038/s41598-020-64982-6

**Published:** 2020-05-20

**Authors:** Piyal Karunarathne, Anna V. Reutemann, Mara Schedler, Adriana Glücksberg, Eric J. Martínez, Ana I. Honfi, Diego H. Hojsgaard

**Affiliations:** 1 Department of Systematics, Biodiversity and Evolution of Plants, Albrecht-von-Haller Institute for Plant Sciences, University of Goettingen, Untere Karspuele 2, 37073 Goettingen, Germany; 2 Georg-August University School of Science, University of Goettingen, Goettingen, Germany; 3 Instituto de Botánica del Nordeste (IBONE), Facultad de Ciencias Agrarias, Universidad Nacional del Nordeste (FCA-UNNE), CC209, 3400 Corrientes, Argentina; 4 Programa de Estudios Florísticos y Genética Vegetal, Instituto de Biología Subtropical (CONICET-UNaM), Facultad de Ciencias Exactas, Químicas y Naturales, Universidad Nacional de Misiones, Rivadavia 2370, 3300 Posadas, Misiones Argentina

**Keywords:** Structural variation, Plant evolution

## Abstract

In systems alternating between sexual and asexual reproduction, sex increases under unfavorable environmental conditions. In plants producing sexual and asexual (apomictic) seeds, studies on the influence of environmental factors on sex are equivocal. We used *Paspalum intermedium* to study environmental effects on the expression of sexual and apomictic developments, and on resulting reproductive fitness variables. Flow cytometric and embryological analyses were performed to characterize ploidy and reproductive modes, and effects of local climatic conditions on sexual and apomictic ovule and seed frequencies were determined. Seed set and germination data were collected and used to estimate reproductive fitness. Frequencies of sexual and apomictic ovules and seeds were highly variable within and among populations. Apomictic development exhibited higher competitive ability but lower overall fitness. Frequencies of sexual reproduction in facultative apomictic plants increased at lower temperatures and wider mean diurnal temperature ranges. We identified a two-fold higher fitness advantage of sexuality and a *Tug of War* between factors intrinsic to apomixis and environmental stressors promoting sexuality which influence the distribution of sex in apomictic populations. This points toward a crucial role of local ecological conditions in promoting a reshuffling of genetic variability that may be shaping the adaptative landscape in apomictic *P. intermedium* plants.

## Introduction

Though many eukaryotes, especially protists, have given it up, the earliest common eukaryote ancestor was sexual. Sexuality promotes the creation of genetically variable and physiologically flexible organisms capable of coping with spatial and temporal environmental heterogeneity. In contrast, asexual reproduction, either by vegetative propagation or by the formation of genetically unreduced gametes that produce new generations of organisms parthenogenetically (apomixis), can rapidly exploit favorable habitats. Taxa from various phylogenetic groups are capable of sexual and apomictic reproduction. In these organisms, stresses associated with changing environmental conditions often suppress asexual reproduction while inducing sexual reproduction^1–3^. Many eukaryotes are capable of producing offspring either sexually or asexually in the same or different generations^4,5^. Gametophytic apomixis is a common form of apomixis in higher plants. Here, the sexual process is terminated either before, during or shortly after meiosis, and it is replaced by unreduced gametophyte (embryo sac) formation. In these gametophytes, the unreduced egg cell develops a clonal embryo parthenogenetically, and the development of the endosperm may or may not require fertilization^5^. Such developmental changes are associated with particular genetic and epigenetic backgrounds that fix apomixis transgenerationally^6–8^, except perhaps for the very early stages in the evolution of a new lineage^9^. Thus, apomixis in plants is not cyclical (see possible cases of reversals to sexuality in^10^), but facultative, meaning that it is expressed at different levels in apomictic individuals. Variable rates of sex (mostly low) have been found in different apomictic plant species^11–16^. Residual sex in apomictic plants plays a relevant role helping clonal organisms to purge deleterious mutations^17^ and creating the genotype variability needed to fractionate the ecological niche space and use local resources (Frozen Niche Variation Model^18^). Selection of new apomictic genotypes adapted to novel environmental conditions is crucial in promoting niche shifts and departures from areas of ecological competition with sexual counterparts (e.g.^19^). Moreover, apomixis provides a colonizing advantage via uniparental reproduction compared to sexual allogamous pairs (Baker’s Law^20^), endorsing range expansions and patterns of reproductive mode distributions known as geographical parthenogenesis^21,22^.

Thus, besides residual sexuality has a central evolutionary role on the maintenance of apomictic plant lineages, little is known about its interaction with and modulation by environmental signals. There is a lack of studies on natural populations, and experimental analyses are equivocal. Schinkel *et al*.^16^ collected population-level data on reproductive mode variations in the facultative apomict *Ranunculus kuepferi*, but the data was organized into three reproductive categories (*i.e*. sexual, facultative apomicts, obligate apomicts) and hence they refrained from a direct evaluation of the influence of environmental factors on proportions of sexuality. While most experimental studies on individual plants demonstrate an influence of different stressors on observed proportions of sexual and apomictic ovules, they have not analyzed or have failed finding any influence on proportions of seeds and progenies. For example, studying plants of *Dichanthium aristatum* artificially grown in a range of climatic conditions throughout 27 degrees latitude, Knox^23^ revealed an association between photoperiods prevailing during the development of inflorescences and the proportion of apomixis. In a transplant experiment, Quarin^24^ found a similar quantitative response between the expression of apomixis in ovules of *Paspalum cromyorrhizon* plants and seasonal variation of daytime. Gounaris *et al*.^25^ exposed apomictic plants of *Cenchrus ciliaris* to a series of inorganic salts on a daily base to observe abnormal features in pistils of salt-treated plants, including an increase in the number of sexual embryo sacs. In another study, Mateo De Arias^26^ exposed apomictic and sexual *Boechera* species to drought stress and drought plus heat stresses and found that the frequency of sexual ovules increased significantly compared to plants without stress, but did not observe changes in the frequencies of sexual and apomictic seeds. Similarly, Klatt *et al*.^27^ grew different clones of the apomict *Ranunculus carpaticola* x *cassubicifolius* under a prolonged photoperiod and observed a significant increase in the frequency of ovules with functional meiotic megaspores without a significant increase in sexual seeds. Rodrigo *et al*.^28^ exposed plants of apomict *Eragrostis curvula* to drought stress conditions and showed that, under water deprivation, facultative apomictic plants increased the formation of sexual embryo sacs but showed no influence on numbers of sexual offspring. Therefore, even when varied environmental stressors including heat, drought, light and nutrient availability induce an increase in the expression of sexuality during ovule development, their effects on the formation of sexual offspring are still unclear.

Indirect evidence of a possible influence of environmental factors on rates of sexual versus apomictic seed formation comes from epigenetic studies. By analyzing open pollinated seeds of apomictic *Paspalum simplex* plants exposed to 5’-azacytidine, a demethylating agent, Podio *et al*.^29^ found high levels of cytosine methylation at the apomixis-controlling genomic region in both species, and a significant suppression of parthenogenesis during seed development. Kirioukhova *et al*.^30^ using bisulfite sequencing and *in situ* hybridization, found that locus-specific DNA methylation changes in apomictic *Boechera* cause aberrant imprinting which affects maternal versus paternal activation of genes and may underlie the emergence of parthenogenesis in species of this genus. Even when parthenogenesis is under genetic control separate from apomeiosis and endosperm formation in apomictic plants (for a detailed discussion see^31^), the above studies point to a relevant role of epigenetic regulation of the trait and hence, it could be affected by different environmental stressors in natural populations.

In single ovules of many apomicts, both meiotic and apomictic pathways can run in parallel differing in spatiotemporal controls of developmental steps^32,33^. Flowers of apomictic plants exhibit high asynchronous development and substantial changes in gene expression patterns compared to flowers of sexual plants^34–36^. Hence, modulation of sex during flower development in facultative apomictic plants is seemingly highly sensitive to environmental signals. As the formation of a new offspring goes through several developmental checkpoints (e.g. sporogenesis or gametogenesis in ovules, embryo or endosperm developments in seeds)^37^, plants with facultative apomixis may show variable reproductive outputs and fitness due to differential effects of environmental stressors on the development and competition between meiotic and apomictic pathways within flowers. Studying apomictic *Paspalum malacophyllum* genotypes, Hojsgaard *et al*.^14^ showed that reproductive competition in ovules varies substantially among individuals, but all shared a significant increase in the efficiency of the apomictic pathway to the detriment of the sexual one toward the formation of seeds and offspring.

Hence, understanding how functional sex is environmentally modulated, its prevalence at local and regional scales and its contribution to the relative fitness of facultative apomictic plants will shed light on the causal success of sexuality *versus* asexuality in natural populations.

Here, we analyze levels of functional sexuality in geographically widespread populations of a facultative apomictic species under a variety of ecological conditions, and their relative contribution to plant fitness. We aim at (1) assessing the expression of sexuality in facultative apomictic populations, (2) evaluating the efficiency of both meiotic and apomictic pathways in the formation of fertile seeds, (3) examining ecological and environmental factors possibly influencing the expression of sexuality, and (4) analyzing the impact of variable levels of sex and apomixis on maternal fitness at different geographic scales. In order to do so, we used *Paspalum intermedium* Munro ex Morong & Britton, a caespitose perennial grass that grows in marshes and wetlands of South America, and has two cytotypes: self-sterile sexual diploids and self-fertile, facultative aposporous tetraploids^38,39^. Both cytotypes co-occur in different arrangements (*i.e*. allopatry, sympatry, and parapatry) adapted to considerably different ecological settings and out-competing each other in their main distribution zones^19^. Thus, *P. intermedium* is a suitable model to study how environmental heterogeneity influences the expression of sexuality and plant fitness under diverse reproductive modes and ecological setups.

## Results

### Ploidy and reproductive mode evaluation of *P. intermedium* cytotypes

**Figure 1:**
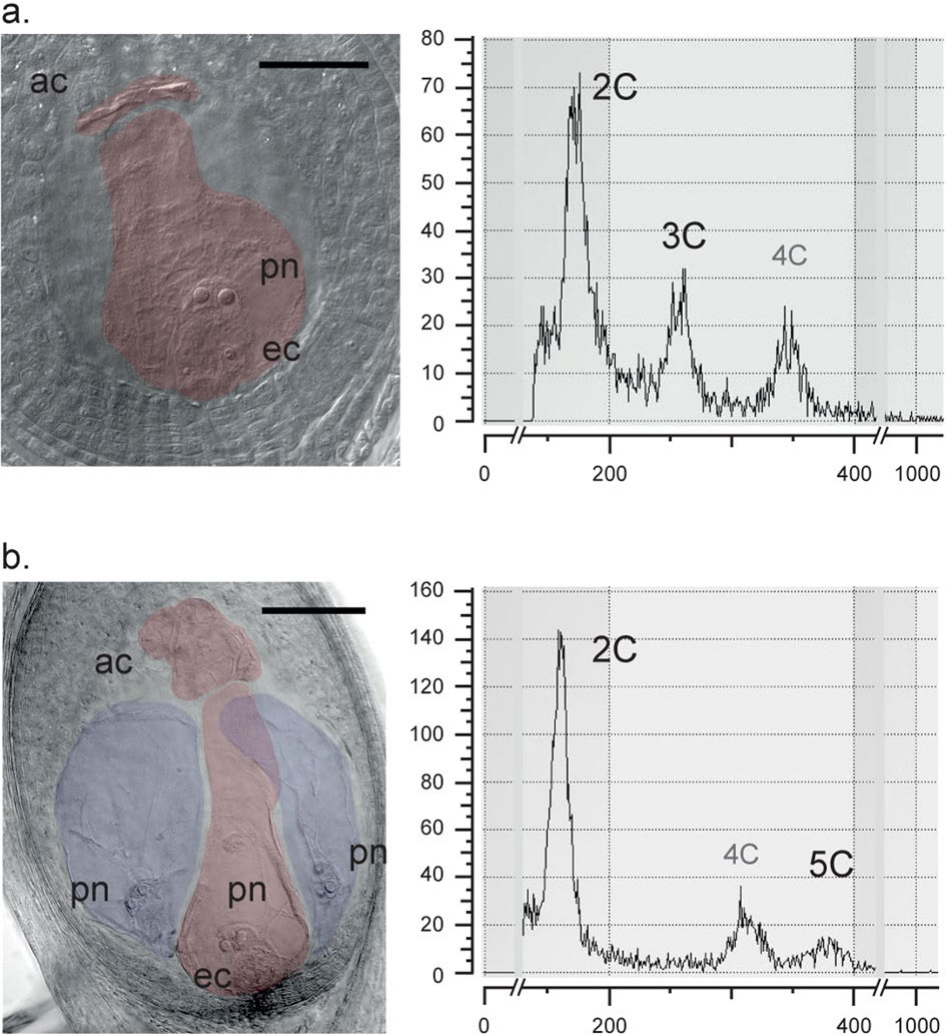
Reproductive analyses in tetraploid *Paspalum intermedium* plants. (**a**) Microscopic image of an ovule carrying a single MES; (**b**) Flow Cytometry histogram of sexual seed having a diploid embryo peak (2C) and a triploid endosperm peak (3C); (**c**) microscopic image of an ovule carrying one MES (red) and two AES (blue), with different spatial (MES sited toward the micropyle) and anatomical (AES lack antipodal cells) features; (**d**) Flow Cytometry histogram of an apomictic seed having a diploid embryo peak (2C) and a pentaploid endosperm peak (5C). *ac*: antipodal cells; *ec*: egg cell; *pn*: polar nuclei in the central cell. The bar represents 50 μm.

Reproductive pathways were assessed in 1181 mature ovules of *P. intermedium* (with an average of 17 ovules per individual and 51 ± 7.3 per population) from 16 pure tetraploid populations, four pure diploid populations and three mixed-ploidy populations (Table 1; Suppl. Table 1). Ovules analyzed during male meiosis showed apomixis is initiated from a nucellar cell surrounding the germline. Meiotic embryo sacs (MES) and apomictic embryo sacs (AES) were differentiated by their anatomical characteristics (Fig. 1).

**Table 1:** Proportion of sexual (meiotic) and apomictic reproductive pathways in ovules at blooming (mature embryo sacs) and seed stages of the studied *P. intermedium* populations.

Collection code	Ploidy (x)*	ES proportions		Seed proportions		χ^2§^	*p*-value^†^
		Meiotic	Apomictic	Sexual	Apomictic		
Hojs402	4	0.161	0.839	0.169	0.831	0.0530	0.817
Hojs403	4	0.318	0.591	0.068	0.932	34.906	**<0.001**
Hojs404	4	0.333	0.667	0.076	0.924	51.020	**<0.001**
Hojs405	4	0.385	0.615	0.100	0.900	34.305	**<0.001**
Hojs409	4	0.267	0.733	0.061	0.939	21.767	**<0.001**
Hojs410	4	0.327	0.673	0.263	0.737	1.8500	0.174
Hojs414	4	0.050	0.950	0.067	0.933	0.1210	0.780
Hojs415	4	0.250	0.750	0.143	0.857	6.1180	**<0.05**
Hojs424	4	0.333	0.667	0.129	0.871	18.810	**<0.001**
Hojs445	4	0.433	0.567	0.096	0.904	46.148	**<0.001**
Hojs453	4	0.466	0.534	0.167	0.833	31.260	**<0.001**
Hojs455	4	0.383	0.617	0.225	0.775	10.511	**<0.001**
Hojs465	4	0.500	0.500	0.076	0.924	71.978	**<0.001**
Hojs475	4	0.396	0.604	0.184	0.816	18.844	**<0.001**
Hojs478	4	0.063	0.938	0.037	0.963	1.1410	0.286
Hojs471	4	0.360	0.640	0.172	0.828	15.357	**<0.001**
Hojs470	2,3,4	0.725	0.275	0.333	0.667	76.955	**<0.001**
Hojs456	2,4	0.444	0.556	0.235	0.765	17.644	**<0.001**
Hojs468	2	0.980	0.000	1.000	0.000	—	—
Hojs422	2	0.990	0.000	1.000	0.000	—	—
M26	2	0.990	0.000	1.000	0.000	—	—
M31	2	0.990	0.000	1.000	0.000	—	—

*According to this study and^19^. ^§^Chi-squared values for observed proportions of sexual and apomictic pathways; ^†^significance values are in bold.

All ovules from diploid plants were bearing single MES except for one ovule which had twin MES. In tetraploid plants, three kinds of ovules were found: i) ovules carrying only MES, ii) ovules carrying only AES, and iii) ovules carrying MES + AES. In ca. 40% of ovules with AES or MES + AES more than one AES were observed. Aborted ES or in abortion were observed, and in a few ovules (ca.<1%) no ES was detected. The overall percentages of MES and AES for all tetraploid individuals were 34.5% and 65.3%, respectively. Nevertheless, the values of MES and AES varied immensely among all the studied populations (Table 1).

Flow cytometry histograms were generated for over 1500 seeds of *P. intermedium* (averaging 75 seeds per population) from 14 pure tetraploid populations, four pure diploid populations, and three mixed populations. Histogram analyses revealed all seeds from diploid plants were of sexual origin (Fig. 1a) while among tetraploid plants showed wide variation in the proportion of sexual and apomictic seeds (Fig. 1b, Table 1), with overall values of ca. 18% sexual and 82% apomictic seeds. Seeds formed after fertilization of an unreduced egg-cell (i.e. B_III_ seeds) were no found, indicating a strict coupling of apomictic embryo sac formation and parthenogenesis in all populations.

Embryology and flow cytometric approaches showed that all diploids are exclusively sexual, and tetraploids are facultatively apomictic.

### Reproductive parameters, efficiency, and competition between reproductive pathways

The average value of sexual reproductive potentials was 0.485 ± 0.048 (ranging between 0.062–0.85 among populations), and 0.794 ± 0.036 (ranging 0.40–1.00 among populations) for apomixis (Table S2). A high Pearson correlation (α = 0.05 as default) was observed between the sexual reproductive potential and ovules with simultaneous MES and AES (*r* = 0.70), plus a high positive correlation between apomictic potential and the presence of more than one AES per ovule (*r* = 0.60) and a negative correlation to MES (*r* = −0.77) (Table S2). Similarly, the sexual reproductive potential was negatively correlated to percentages of apomixis in populations (*r* = −0.89) (Table S2). Therefore, the data suggest that multiple AES are a proxy for the penetrance of asexuality and depletion of meiosis in natural populations.

The observed proportions of meiotic (and apomictic) reproductive pathways at ovule and seed stages showed a significant difference among populations (paired *t*-test *p* = 0.009). Significant differences were also found in comparisons within populations (chi-squared test χ^2^ < 6.11, *p* < 0.013 in all but two populations, Hojs402 and Hojs478; Table 2). The overall proportion of sexual seeds exhibits a significant reduction from the expected 38.2% to the observed 15.3% (*p* = 0.001, χ^2^ = 9.847), while the proportion of apomictic seeds showed a substantial increase from the expected 61.8% to the observed 84.7% (*p* = 0.049, χ^2^ = 3.594) (Table 2). At the population level, most differences between expected and observed values were significant (Table 2), the highest being 42.4% (Hoj465; Table 2), while the lowest was 0.8% (Hoj402; *p* = 0.907; Table 2). A reduction in the reproductive efficiency of the sexual pathway between ovules and seeds was observed in all the studied populations, ranging from 0.981 to 0.152 (Table 2). Contrary, an increase ranging from 1.004 to 2.072 was observed in apomictic pathways (Table 2). These increases of apomictic efficiencies showed no correlation to the number of ovules with MES + AES (*r* = −0.08) but were negatively correlated to the number of ovules with multiple AES (*r* = −0.57), likely because the higher the penetrance of apomixis, the lower it is the potential for sexuality and smaller the gap to increase the efficiency of the asexual pathway. The reproductive efficiency of sexual or apomictic pathways showed a low correlation to the formation of sexual (*r* = 0.41) or apomictic (*r* = −0.24) seeds, respectively. However, when considering the sexual reproductive potential and efficiency of each population together, we found a high correlation to the proportion of sexual seeds (*r* = 0.96) formed in different populations, while a similar analysis for apomixis showed moderate correlation (*r* = 0.50). The data suggest that the reproductive potential of each pathway and its efficiency depends on the geographic location, which has a more significant impact on the formation of sexual than asexual seeds. When plotted against mean diurnal temperature ranges (MDR), sexuality benefit more than apomixis by increasing MDR (Figure S1; see details in the section *Climatic variation, spatial incidence of reproductive pathways and model predictions*).

**Table 2:** Analysis of reproductive pathway competition and efficiency during formation of female gametophytes, double fertilization and development of functional seeds among polyploid populations of *P. intermedium*.

Collection code	Reproductive mode proportions				*χ*^*2*^	*p-*value^†^	Reprod. efficiency	
	Sexual		Apomictic				*Sexual*	*Apom*.
	*exp*.	*obs*.	*exp*.	*obs*.				
Hojs402	0.173	0.169	0.827	0.831	0.0110	0.916	0.981	1.004
Hojs403	0.415	0.068	0.585	0.932	49.597	**<0.001**	0.164	1.594
Hojs404	0.387	0.076	0.613	0.924	38.126	**<0.001**	0.196	1.507
Hojs405	0.401	0.100	0.599	0.900	37.719	**<0.001**	0.249	1.503
Hojs409	0.307	0.061	0.693	0.939	28.445	**<0.001**	0.198	1.355
Hojs410	0.356	0.263	0.644	0.737	3.7730	**<0.05**	0.740	1.144
Hojs414	0.093	0.067	0.907	0.933	3.0140	**<0.05**	0.719	1.029
Hojs415	0.318	0.143	0.682	0.857	14.121	**<0.001**	0.450	1.256
Hojs424	0.376	0.129	0.624	0.871	26.003	**<0.001**	0.342	1.397
Hojs445	0.448	0.096	0.552	0.904	50.104	**<0.001**	0.215	1.636
Hojs453	0.469	0.167	0.531	0.833	44.751	**<0.001**	0.355	1.569
Hojs455	0.429	0.225	0.571	0.775	16.989	**<0.001**	0.525	1.358
Hojs456	0.463	0.235	0.537	0.765	20.908	**<0.001**	0.509	1.423
Hojs465	0.500	0.076	0.500	0.924	71.910	**<0.001**	0.152	1.848
Hojs470	0.678	0.333	0.322	0.667	54.520	**<0.001**	0.491	2.072
Hojs475	0.400	0.184	0.600	0.816	19.440	**<0.001**	0.459	1.361
Hojs478	0.063	0.037	0.938	0.963	1.0750	0.300	0.593	1.027
Hojs471	0.407	0.172	0.593	0.828	22.882	**<0.001**	0.423	1.396

^†^Level of significance are in bold; *exp*.: expected proportions; *obs*.: observed proportions; Reproductive efficiency values = 1 reflects an equal efficiency between the expected and observed reproductive values for each reproductive pathways, values < 1 indicates inferior efficiency and values > 1 superior efficiency.

In mixed-ploidy populations, tetraploids showed considerably different proportions of sexuality and apomixis compared to those in tetraploids of pure populations. While the average number of ovules with MES was 45% (ranging from 33%-51%) in mixed populations, it was significantly different in pure tetraploid populations showing an average of 34.1% (ranging from 7%-41%). At the seed stage, the same mixed populations showed a proportion of sexual seeds (21%) closer to that of pure tetraploid populations (ca. 18%).

### Fitness variation in diploid and tetraploid cytotypes

Fecundity assessments showed no significant differences in the average number of spikelets produced per inflorescence between diploid and tetraploid populations (4805.18 florets in diploids, 4348.87 in tetraploids; Table 3, Table S3). However, percentages of full seeds produced by diploids were twice that of tetraploids (32.61% in diploids, 15.83% in tetraploids; Table 3, Table S3). Fertility measured by germinability tests also showed no significant differences in both diploid and tetraploid individuals and populations (0.74 ± 0.13 and 0.79 ± 0.12, respectively; Table 3, Table S3). Consequently, diploids have a two-fold higher reproductive fitness (*f*_2*x*_ = 0.276) compared to tetraploids (*f*_4*x*_ = 0.135).

**Table 3:** Analysis of female fitness of diploid and tetraploid populations assessed in Northern, Central and Southern distribution ranges of the species.

	Seed set		Spikelets^§^		Inflorescences^†^		Fecundity		Germinability		Fitness
	%	SE	*n*	SE	*n*	SE	*prop*.	SE	*prop*.	SE	*prop*.
**OVERALL FITNESS**											
2×	32.6	3.15	4805.2	360.6	39.2	5.13	0.37	0.06	0.739	0.025	0.276
4×	15.8	1.51	4348.9	296.8	31.9	2.70	0.17	0.03	0.791	0.017	0.135
**REGIONAL FITNESS**											
**Northern**											
2×	31.2	14.97	5261.3	436.6	35.8	6.49	0.37	0.07	0.702	0.041	0.255
4×	18.7	8.32	4349.3	412.4	34.2	5.99	0.18	0.03	0.760	0.029	0.137
**Central**											
2×	29.1	4.74	4381.7	556.2	42.4	7.81	0.38	0.10	0.774	0.026	0.296
4×	18.1	3.25	4555.9	537.5	33.9	4.67	0.19	0.06	0.742	0.028	0.154
**Southern**											
2×	—	—	—	—	—	—	—	—	—	—	—
4×	14.4	2.05	4219.8	461.5	32.6	4.59	0.17	0.04	0.829	0.028	0.135
**Mixed population (central region)**											
2×	10.2	1.26	3934.4	691.1	14.7	2.91	0.04	0.00	0.829	0.087	0.031
3×	39.5	12.65	2590.0	1586.0	8.0	2.00	0.06	0.03	0.710	0.000	0.039
4×	15.0	7.62	4640.4	745.1	27.6	10.81	0.29	0.17	0.704	0.063	0.293
**Pure population (central region)**											
2×	37.0	5.69	4026.0	599.8	58.7	8.22	0.55	0.11	0.787	0.020	0.435
3×	—	—	—	—	—	—	—	—	—	—	—
4×	20.0	3.39	4433.7	590.5	36.9	4.77	0.22	0.06	0.768	0.024	0.174

^§^number of spikelets per inflorescence; ^†^number of inflorescences per individual; SE: Standard Error.

The regional evaluation of fitness components for different populations (Table 3) indicates that tetraploids have lower fecundity in Southern areas (14.4%) than in Northern (18.7%) and Central areas (18.1%), while diploids in these areas maintain similar values (31.2% and 29.1%) (Table 3). Interestingly, the pure populations of both cytotypes in the Central region showed the highest values of fecundity (37.0% in diploids, 20.0% in tetraploids) and fitness (*f*_2*x*_ = 0.435, *f*_4*x*_ = 0.174). In mixed-ploidy populations, diploid individuals showed the lowest fitness values (*f*_2*x*_ = 0.031) compared to all diploid populations, whereas local tetraploids surpassed the fitness of diploids by almost 10 folds (*f*_4*x*_ = 0.293) (Table 3). The only triploid found in a mixed-ploidy population surprisingly showed the highest seed set value (39.55%) observed among all cytotypes, although its fitness was very low (*f*_3*x*_ = 0.039) due to a reduced number of inflorescences and low germinability compared to other populations (Table 3).

### Climatic variation, spatial incidence of reproductive pathways and model predictions

**Figure 2:**
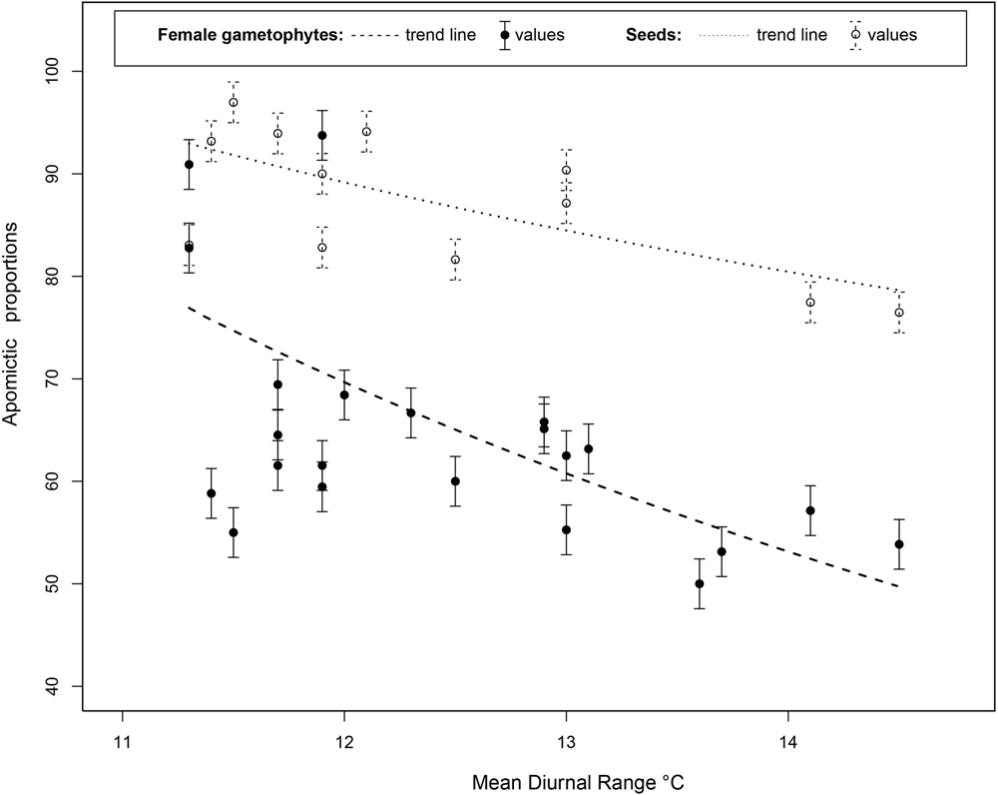
Plot showing the GLM fitted value lines of apomictic percentages of both embryo sacs and seeds in all the studied *P. intermedium* populations.

Correlation analysis of bioclimatic data and geographical distributions of cytotypes showed a significant differentiation for 15 environmental variables and indicated unique ecological and climatic preferences, mainly based on temperature, precipitation and radiation-related variables (for more details see^19^). The influence of these environmental conditions on developmental steps (*i.e*. ovules or seeds) driving the formation of new offspring was evaluated. Initial scatter plots of each environmental variable and proportions of apomictic and sexual pathways showed no visible patterns for all but five variables. At ovule stage, a Pearson test confirmed a high correlation between reproductive pathways and Mean Diurnal Range (MDR) (*r*_sex_ = 0.68, *r*_apo_ = −0.70; Table S4), Temperature Annual Range (*r*_sex_ = 0.69; *r*_apo_ = −0.69), Precipitation of Driest Quarter (*r*_sex_ = −0.67; *r*_apo_ = 0.67), and Precipitation of Coldest Quarter (*r*_sex_ = −0.67; *r*_apo_ = 0.67) (Fig. 2; Table S4). A Pearson’s test for seed stage showed a moderate-to-high correlation between reproductive pathways and MDR (*r*_sex_ = 0.56; *r*_apo_ = −0.52), and Precipitation of Driest Month (*r*_sex_ = −0.52; *r*_apo_ = 0.52) (Fig. 2; Table S4). Thus, dissimilar environmental factors have a differential effect on reproductive alternatives at each developmental stage. The only variable showing a consistent significant effect on reproductive output in both ovules and seeds was MDR; therefore, we focused our following analysis on this environmental factor. The observed correlations to environmental variables were always inversely proportionate between reproductive pathways. Two other bioclimatic variables (Bio5 and Bio8) showed strong associations to MDR (*r* = 0.81 and *r* = 0.78, respectively; Table S4) along with moderate correlation to reproductive proportions at developmental stages (e.g., *r* = −0.306 for Bio5, *r* = −0.452 for Bio8 in ovules).

**Figure 3:**
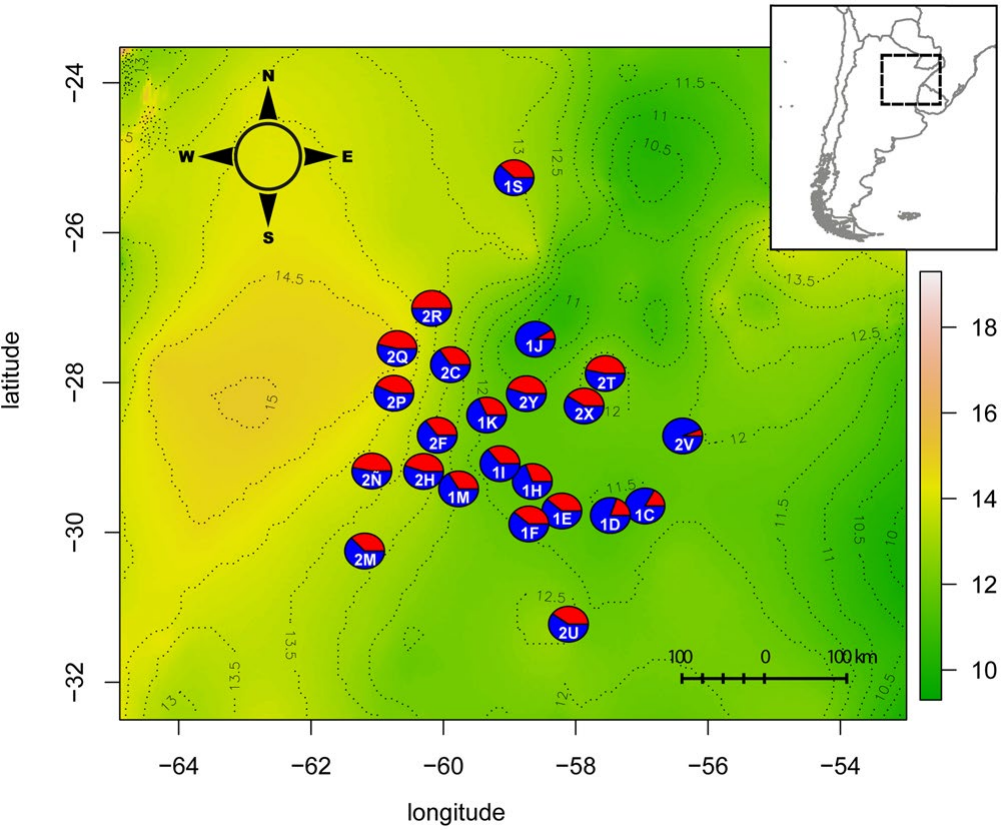
Map depicting the geographic variation of meiotic and apomictic ES percentages in different *P. intermedium* populations plotted against the MDR zones (contour lines demarcate variation zones; temperature ranges are given in Celsius). Red pies represent meiotic proportions; blue pies represent apomictic proportions.

We performed a Generalized Linear Model (GLM) with Gaussian inverse link on MDR data and the proportions of apomictic and meiotic reproductive pathways at two developmental stages. The overall values showed a negative relationship between MDR and the occurrence of AES (*t* = 4.18, *p* = 0.0006) and apomictic seeds (*t* = 2.324, *p* = 0.03), and a positive influence by MDR on the meiotic pathway (*t* = −4.18, *p* = 0.0006). Among-population analyses showed a significant influence of MDR on the proportion of AES and apomictic seeds for all cases, with a stronger effect at median MDR values (GLM *p* = 0.013) and lower influence at higher MDR values (Fig. 3). This indicates that reproductive modes are sensitive to and modulated by the environmental conditions. Therefore, for a better perspective, a nonlinear function that explains the observed response of meiotic and apomictic pathways to MDR was formulated (Fig. S2; Material S1).

**Figure 4:**
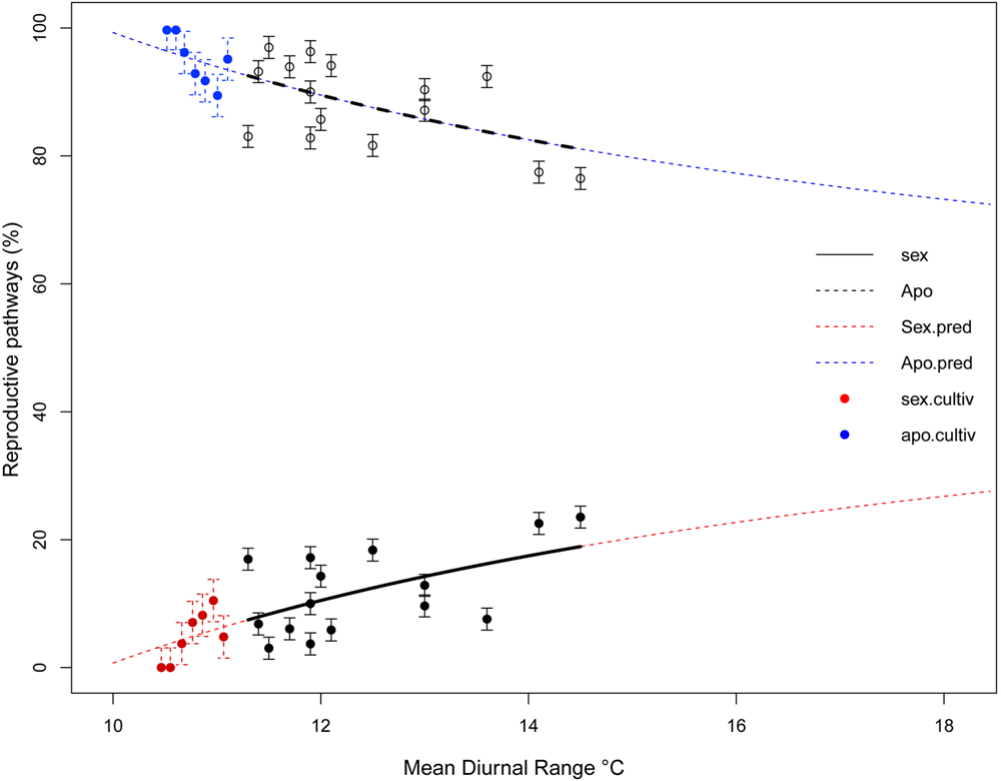
Modelled distribution of reproductive pathway proportions at seed stages. Values of sexuality and apomixis gathered from natural populations are in black. Model projections of predicted mean values for sexuality and apomixis are depicted as red and blue dotted lines, respectively. Values of sexuality and apomixis from common garden experiments corresponding to the MDR zone of 11.1 °C ± 0.2 are in color.

In order to evaluate the accuracy of our model, we used two approaches. First, proportions of sexual and apomictic pathways predicted by the GLM for MDR values beyond the observed range were compared with proportions obtained from common garden experiments (see Fig. 4). Reproductive proportions from individuals cultivated in an area with an MDR of 11.1 °C (no natural population was found at this MDR) showed a range of meiotic and apomictic proportions between 0.00–0.11 (mean value = 0.049 ± 0.014) and 0.89–1.00 (mean value = 0.95 ± 0.014), respectively (Table S5). A *t*-test showed no significant difference (*t* = 1.064, *df* = 6, *p* = 0.328) and a high level of fit between the experimentally obtained reproductive proportions and predictions (Fig. 4) validating the model and the observed environmental modulation on reproduction.

In the second approach, we searched for populations sharing similar reproductive sexual potential located in different MDR zones. If our model is correct, the reproductive efficiency of the sexual pathway in populations at broader MDR should be higher than those at narrow MDR. We identified three groups of populations each having similar sexual reproductive potential (group_1_ = 0.41 ± 0.01, group_2_ = 0.53 ± 0.01, group_3_ = 0.65 ± 0.02) from different MDR zones (*n* = 3, *n* = 4, and *n* = 5, respectively). Except for the first case where a subtle trend was observed (Fig. S3a), likely because it has only three populations spanning a narrow range of MDR zones (between 11.7 °C–12.5 °C), the other two groups spanning through wider ranges (between 11.7 °C–13.7 °C and 11.4 °C–14.5 °C) showed clear positive trends due to increased sexual efficiency and formation of sexual seeds in relation to MDR (Fig. S3b,c).

### Seasonal changes in meiotic and apomictic frequencies

A paired *t*-test between the mean proportion of MES (and AES) for populations collected at two seasons showed significant differences (meiotic pathway: *t* = −2.4566, *df* = 16.296, *p* = 0.0256; apomictic pathway: *t* = 2.99, *df* = 16.591, *p* = 0.008) indicating a seasonal variation in the expression of apomixis and sexuality. However, the proportion of sexual and apomictic seeds showed no significant variations (*t* = 1.0655, *df* = 12.022, *p* = 0.3076). The changes observed in the reproductive efficiency of each reproductive pathway and developmental stage were not significant between seasons (early season: χ^2^ = 42, *df* = 36, *p* = 0.227; late season: χ^2^ = 56, *df* = 49, *p* = 0.2289). Overall, these results suggest that locally, reproductive modes are sensitive to seasonal changes during ovule development but not during seed formation.

## Discussion

Maintenance of sex in dioecious organisms is often associated with a two-fold cost of producing males^40^. However, relative costs of sexual reproduction are taxon-specific, and sexuality is in various ways a less efficient method of reproduction compared with asexuality^41^. In *Paspalum intermedium*, the expression of sexual and apomictic pathways varies highly among populations and is influenced by environmental factors, but asexuals do not show a fitness advantage compared to sexuals, as it would be expected.

### Reproductive variability in *Paspalum intermedium*

The genus *Paspalum* displays a large variation in reproductive systems^39^. As most studied apomictic systems in angiosperms, many *Paspalum* species including *P. intermedium* show a reproductive dimorphism linked to different chromosomal races. Diploid cytotypes are self-sterile obligate sexuals and tetraploid cytotypes are self-fertile facultative apomicts, as previously reported^19,38^. The type of apomixis in *P. intermedium* is apospory, meaning that sexual and asexual reproductive pathways develop from independent cell types. The germline produces meiotic spores and reduced female gametophytes, whereas the apomictic pathway develops from somatic nucellar cells surrounding the germline and produces unreduced gametophytes. In agreement with reproductive studies on individual plants from different species^42–47^, our population level analysis covering most of *P. intermedium*’ geographical distribution shows that diploids have a highly stable sexual reproductive mode while polyploids show variable incidence of both sexuality and apomixis throughout their distribution range. We found levels of sexuality and apomixis (*i.e*. facultativeness) in ovules ranging from 6–68% and 32–94% respectively, and similarly, the variation ranged from 3–33% for sexual and 67–96% for apomictic seeds. Additionally, our analysis revealed that the expression of sexuality and apomixis in tetraploid *P. intermedium* plants is, to a certain extent, geographically structured (see details below). So far, all studies on facultative apomictic plants have suggested that allocation of resources to sexual or asexual seeds is determined collectively by genotype-by-environment interactions^14,48,49^ although no study has tested this using *in situ* reproductive analysis and climatic data.

### Efficacy of the apomictic pathway excels at the cost of a depleted fitness

Despite the observed high variation on reproductive proportions among *P. intermedium* populations (present study^50^), apomixis and its reproductive efficiency increased significantly in most cases at the expenses of sexuality, and yet, the fitness of apomicts was dramatically lower than sexuals. Many spikelets (63% of the total) in different individuals harbored two or more apomictic embryo sacs inside the same ovule, and with the absence of B_III_ individuals in populations together suggest a strong penetrance of the trait which may explain its higher reproductive efficiency. Another factor that might provide an advantage to the apomictic pathway over the sexual one is its higher ploidy. Selection is more effective in eliminating deleterious recessive mutations in haploid organisms than diploids because of masking effects^51^. While no chromosomal reduction occurs in apomictically derived gametophytes, meiotic gametophytes are haploid and more likely to expose deleterious mutations and developmental problems. Yet, since plants are tetraploids, even haploid gametophytes have at least two copies for each locus and thus, masking effects are expected.

Other relevant factors likely influencing the efficiency of reproductive pathways are developmental timing and space competition within the ovule. The orientation of embryo sacs within the ovule is not random^14,52^. AESs are often dislocated toward the chalazal zone and closer to the funiculus, having direct access to resources from the sporophytic tissue. Intercellular communication by vesicle trafficking between spatially separated cells is crucial for the establishment and development of ovular components and polarity^53^. Due to its spatial localization, AESs might interfere with communication between cells and develop faster by capturing resources more efficiently, likely enhanced by its higher ploidy and independence from fertilization. Conversely, MESs had a well-developed egg-apparatus and synergid cells with a filiform apparatus well inserted in the micropylar end of the ovule, conveniently positioned for pollen tube access. Yet, the development of MESs into sexual seeds was drastically reduced in the studied populations. The observation of a strict association between apomixis efficiency and the number of ovules with MES + AES indicates that the sexual pathway in ovules of *P. intermedium* was handicapped and likely failed to form seeds in most cases. A similar observation was reported for five *Paspalum malacophyllum* genotypes which showed a depletion of sexuality that started in ovules, increased at seed stage and was complete in adult progenies^14^.

Further piling on the developmental restriction of meiotic pathways, the genetic nature of the trait is likely playing a role. Apomixis overlays the sexual program^54^ causing gene de-regulations and destabilizing meiosis. Massive up- and down-regulation of genes during megaspore and embryo sac formation characterize apomictic ovules of all studied species in different plant genera, including *Boechera* spp.^34^, *Hieracium* spp.^55^, *Ranunculus* spp.^35^, *Hypericum* spp.^56^, *Pennisetum* spp.^57^, and *Paspalum* spp.^36,58^. In *P. intermedium,* the observed developmental constraints likely associated to the genetic changes inherent to apomixis are destabilizing the sexual development and cause the observed upsurge of aborted ovules and seed formation failure. The consequence is a severe reduction in the fitness of tetraploid apomicts compared to diploid sexuals. In hermaphroditic species where a cost of male production is absent, genome dilution imposes a cost upon the sexual lineage, and an initial 3/2-fold advantage for the asexual one^41^. Hence, apomicts should outcompete sex. Contrary, we observe an overall fitness disadvantage in hermaphroditic apomicts of *P. intermedium*. Comprehensive evaluations of sexual *versus* apomictic plant fitness are scarce, and diverse studies had reported higher or lower seed set by obligate sexual individuals compared to apomictic individuals (e.g. *Ranunculus kuepferi*^16^). In *P. intermedium*, we found a direct link between the incidence of apomixis, ovule abortion and reduced fitness in most natural populations. The only exception was found in populations of sympatric diploids and polyploids, with the apomicts exhibiting a remarkable fitness advantage. This suggests that apomicts may capitalize asexuality benefits during invasion of sexual populations rather than during maintenance. The most likely explanation for this observation is unidirectional interploid introgression. *P. intermedium* is wind-pollinated and lacks pre-mating barriers. Apomictic embryo sacs are recalcitrant to hybridization as the unreduced egg-cell develops parthenogenetically, and restrictions on parental genome contributions to endosperm development are relaxed^59,60^. Even when penetrance of parthenogenesis may vary owing to epigenetic regulation^29,30^, B_III_ individuals were not found in populations of this species. Since *P. intermedium* is a pseudogamous apomict (*i.e*. central cell fertilization is needed for seed development), in mixed populations, egg-cell fertilization in apomict ovules is blocked by parthenogenesis without affecting the fertilization of the central cell and the development of a functional seed. Conversely, introgression of pollen from tetraploids into sexual diploids creates triploid zygotes, central cells with incompatible maternal-to-paternal contributions (*i.e*. triploid block), and cause aberrant seed development^61^. Consequently, unidirectional introgression in mixed-ploidy populations of *P. intermedium* is likely increasing the number of ineffective matings and non-viable progeny in diploids, with dramatic consequences on plant fitness. Experimental crossings will shed light on this hypothesis.

### A reproductive *Tug of War*: environmental stimuli versus genetic setups

Flowering is controlled by environmental factors^62^. Endogenous genetic components and an intricate network of regulatory mechanisms such as photoreception, circadian clock regulation, growth regulator synthesis, chromatin structure, response to low temperatures, etc. sense environmental conditions and play important roles determining flowering-time^63^. Adaptive responses to cold seasonal climates (including cold acclimation, freezing tolerance, endodormancy, and vernalization) point to an evolutionary lability of such traits and a potential role for local adaptation in response to climate change^64^. The relevance of such lability is exemplified by the observed niche transition that enabled the evolution of seasonal cold tolerance within the Pooideae grass family supporting its extensive radiation within temperate regions^65^.

Intrinsically associated with flowering in angiosperms is sexuality, the formation of haploid gametes by meiosis and diploid offspring after syngamy. In facultative apomictic plants, apomixis emerges as a parallel alternative to sexuality, wherein both sexual (meiotic) and apomictic developmental programs can be simultaneously activated and compete within the ovule to produce a seed^14^. In these apomicts, sexuality is the default reproductive mode^66^, and apomixis behaves as dominant over sexuality depleting meiotic genes and reprogramming transcriptional responses to stress conditions^67^. In *Paspalum* spp. and other apomictic grasses, the genetic factors responsible for apomixis are located in a large chromosomal region inherited as one unit^57,68,69^. Sequence-level analyses within the apomixis locus shows frequently interrupted genes^70^, and gene expression studies point toward a genetic reprogramming that affects the expression of a variety of genes, including meiotic genes, transcription factors, stress-associated genes^36,58,67,71^ and genes needed for the emergence of apomixis during ovule development^72^. Therefore, in facultative apomictic grasses, apomixis is a leading developmental mechanism superimposed over the sexual program. Despite its dominant inheritance, apomixis in *P. intermedium* displayed complete penetrance but was geographically structured. Incomplete penetrance and variable gene expressivity has been observed in different apomictic species^73^. In facultative apomictic *P. intermedium*, the interaction between apomixis factors embedded within the particular genomic (sexual) background of each clone and the environmental conditions are likely shaping the variable incidence of each reproductive mode as a whole (i.e. sexual or apomictic) rather than influencing individual components of reproduction (i.e. gamete formation, fertilization, embryo and endosperm developments). Even though previous studies using different experimental setups have signaled an effect of temperature^26,74,75^, water availability^28^ and photoperiod^24,27,76^ on sexuality, no conclusive evidence has been found from natural conditions.

Our pioneering analysis using *in situ* population-level data (present study;^50^) show significant correlations between the occurrence of sexual ovules and seeds and different environmental factors within facultative polyploids of *P. intermedium*. In particular, the seasonal variation of daily temperature was found to be significantly associated with sexual reproductive outputs of populations. A change in the mean diurnal range can induce a stress response and changes in physiology and biosynthesis pathways during flower development^77,78^. Adaptive evolution of low-temperature-induced stress responses is relevant for adaptation to cold habitats in grasses^79^. In asexual plants with reduced genetic and genotype variability, higher frequencies of sex observed in apomictic populations exposed to colder and wider temperature ranges may have an important role facilitating the local adaptation of clonal populations.

Combined with the results from the common garden experiments, the data depict an environmental modulation of sex in *P. intermedium* populations, locally and regionally, and suggest a developmental *tug of war* between environmentally inducible meiosis and genetically dominant apomixis, thus making the most out of the reproductive season. Maternal investment is expected to allocate enough resources among offspring to maximize plant’s fitness^80^. However, apomictic polyploids of *P. intermedium* fail to maximize their fitness even though they can tolerate environmental variability better than sexual diploid parents^19^. An apparent conflict between genetic factors promoting the expression of apomixis and environmental stressors stimulating sexuality is likely the basis for the drastically reduced fitness of facultative apomictic polyploids compared to sexual diploids. Moreover, among tetraploid populations with similar sexual reproductive potential, sexuality is higher and developmentally more efficient toward areas of greater environmental stress. Accordingly, seasonal variations show a significant increase (paired *t*-test *p* = 0.01) in the formation of meiotic female gametophytes in apomictic populations during the drier and warmer season (December-March). Our modelling on population-level data further indicates that full sexuality will never be reached (fixed) in these polyploids, a situation observed in *P. intermedium* as well as in all other studied polyploid apomictic species, for which natural obligate sexual individuals are yet to be recorded^5,81^.

Temporal and spatial changes in the incidence of sexuality in facultative apomicts are likely a dual consequence of the inherent genetic nature of apomixis (deregulated expression of gene networks) and the modulation of sexuality by environmental variables. Such interaction might evolve as a response to transient adaptive pressures, allowing clonal lineages to retain levels of residual sexuality accordingly and keeping pace with surrounding environmental changes by creating new gene combinations able to leverage novel ecological challenges.

## Conclusions

In this study we found a connection between environmental heterogeneity, relative investment in sexual vs. asexual (aposporous) seed formation and plant fitness in the grass species *Paspalum intermedium*. A link between environmental stressors (in particular mean diurnal temperature range) and increased frequencies of sexual (against apomictic) reproduction in natural populations was exposed, and corroborated under experimental conditions by model predictions. Altogether, the data point to a reproductive “tug of war” between genetically dominant apomictic and environmentally sensible sexual pathways, with an overall negative outcome (lower fitness) for the facultative apomictic individuals relative to obligate sexuals. Despite reduced fitness, higher local rates of residual sex in areas of greater environmental stress likely have a functional-evolutionary advantage.

## Materials and Methods

### Plant materials and ploidy levels

*P. intermedium* plants do not propagate vegetatively, and flowering and fruiting occur from late October till early May. Florets (spikelets) are hermaphrodites, exclusively wind pollinated and aggregated in racemes in inflorescences. In total, 39 *P. intermedium* populations covering most of the main geographical distribution area of the species were identified in two field trips during the beginning and end of the flowering season (November-December and March, respectively) (Table S1). Ploidy levels of around 30 individuals per population were analyzed by chromosome counts and flow cytometry^19,50^. A total of 11 pure diploids, 24 pure tetraploids, and four mix ploidy populations were identified (Table S1).

### Common garden experiments

Three to five individuals per population were transplanted to a common environmental setting in experimental gardens at the Faculty of Agrarian Sciences, National University of the Northeast, Argentina. Around 25 plants from seven different populations were also kept under controlled temperature and humidity (24 °C, 10–12 h/day photoperiod, 250 mmol/m2/s light intensity and 80% humidity) inside walk-in climate chambers (YORK^®^ Climate chamber, Johnson Controls, Milwaukee, USA) at the Albrecht-von-Haller Institute for Plant Sciences, University of Goettingen, Germany.

### Reproductive pathway analyses

The reproductive mode of three individuals per population was characterized at two developmental stages using two techniques: embryology in ovules and flow cytometry seed screening (FCSS) (present study^50^).

*Embryological analysis*. Inflorescences of 27 *P. intermedium* populations were collected *in situ* during field explorations, fixed in FAA for 24 hours, transferred to 70% ethanol and stored at 4 °C. Individual flowers were dissected under a Stereomicroscope (Leica M125; Leica Microsystems GmbH, Wetzlar, Germany), ovaries were cleared using Methyl Salicylate^82^ and analyzed using a Differential Interference Contrast (DIC) microscope (Leica DM5500B). A total of ca.100 ovules from randomly selected individuals fixed during male meiosis were analyzed to check the type of gametophytic apomixis (*i.e*. diplospory or apospory). For the evaluation of reproductive parameters including reproductive efficiency, distribution of sexuality and environmental modulation (see details below), 10–20 ovules fixed at anthesis were examined per individual from three individuals per population.

### Flow cytometry seed analysis

Mature seeds from 20 *P. intermedium* natural populations and 6 populations from common garden experiments were collected under open pollination conditions. At least 30 seeds from each individual (around 100 seeds per population) were assessed following the protocol described in Karunarathne *et al*.^19^ (see also^50^). Single seed histograms were produced in a Cube 6 Ploidy Analyzer (Sysmex-Partec GmbH, Görlitz, Germany) and were analyzed using CyView^TM^ data processing software (Sysmex-Partec GmbH). The relative fluorescence of at least 3000 particles (nuclei) from each seed was measured and histogram peaks were assigned to embryo and endosperm tissues. A maximum coefficient of variation (CV) of 5% was accepted for each histogram peak.

### Fecundity (seed set) and fertility (offspring) assessment

#### Seed set

The number of seeds produced throughout the season was used as a surrogate for fecundity^83,84^. Thus, fecundity was estimated as the average number of seeds produced per individual. During flowering, once all spikelets were in anthesis, three to six inflorescences from each individual were bagged using Sulphite-paper crossing-bags (Baumann Saatzuchtbedarf GmbH, Waldenburg, Germany). One month after bagging, the inflorescences were collected and full and empty spikelets (with and without caryopses, respectively) were sorted out in two groups using a 757 South Dakota Seed Blower (SeedBuro Equipment Company, Illinois, USA). Total numbers of full and empty spikelets were estimated by weighing three sets of hundred spikelets from each inflorescence, averaging and extrapolating that value to the total weight of each seed groups per individual. The total number of inflorescences was recorded throughout the flowering season and used to calculate the number of flowers (ovules) and seeds produced by each individual and population.

#### Offspring

Fertility, as the capability to produce offspring^83,85^, was determined by the number of seedlings produced after seed germination tests. Seeds from three individuals per population and a total of 30 populations were sown in sterilized soil and kept under the same light, temperature and water regime. Germination ability was checked every second day for 60 days and used to estimate germinability for each individual and population.

### Reproductive parameters, pathway efficiency, and maternal fitness

Proportions of sexual and apomictic embryo sacs and seeds were used to estimate several reproductive parameters. The (observed) proportions of embryo sacs were estimated as *nm*/(*nm* + *na*) for the meiotic pathway and *na*/(*nm* + *na*) for the apomictic pathway, where *nm* is the total number of ovules with a meiotic embryo sac (MES), and *na* is the total number of ovules with apomictic embryo sacs (AES). A similar formula was used for estimating observed proportions of sexual and apomictic seeds. The expected proportion of sexual and apomictic seeds was calculated as nm + 0.5 nma/nt and na + 0.5nma/nt, respectively, where nma is the number of observed ovules with both meiotic and apomictic pathways. In our analysis, it was assumed that (i) MES and AES develops independently from each other, and (ii) they have the same probability to form a seed. The reproductive potential for sexuality and for apomixis was estimated as *nm*/*nt* and *na*/*nt*, respectively, where *nt* is the total number of ovules (or seeds) analyzed. The efficiency of each reproductive pathway (sexual and apomictic) in tetraploid plants was calculated as the ratio between the observed and the expected proportions of flowers undergoing the meiotic or the apomictic pathway. A paired *t*-test and a standard Pearson’s Chi-squared test were performed to check for significant differences between observed and expected values at both developmental stages.

Analyses of plant fitness were focused on *maternal fitness*. The effect of *paternal fitness* was considered negligible because (1) tetraploid apomicts in *P. intermedium* are self-pollinated, (2) male gametes do not contribute to the formation of parthenogenetic embryos in apomictic seeds, and (3) the maternal genotype and environments are both known to affect offspring performance^86^. Estimation of possible maternal fitness context-dependent effects on rates of self-fertilization or inbreeding depression is not needed as they are skipped by apomictic progenies, and might affect only sexual progenies. Therefore, in *P. intermedium*, fitness estimates based on seed quantity and germinability are expected to reflect plant´s fitness effectively. Measures of differential reproductive success or *maternal fitness* were estimated as a product of *fecundity* and *fertility* values for each individual and population.

### Ecological and seasonal effects and modeling of reproductive modes

Data for ecological/environmental analysis were downloaded from open source databases: 19 bioclimatic variables downloaded from WorldClim (1950–2000; version 1.4^87^, www.worldclim.org), UV-B radiation downloaded from glUV (www.ufz.de/gluv)^88^, photosynthetically active radiation (PAR) data downloaded from Moderate Resolution Imaging Spectroradiometer (MODIS) database;^89^ https://lpdaac.usgs.gov), and cloud cover, frost day frequency, and vapor pressure at ground level downloaded from CGIAR-CSI (www.cgiar-csi.org). The data was downloaded as raster grid files either at 2.5 arc minute resolution or (dis)aggregated to match 2.5 arc minute resolutions. The environmental data for each population was extracted from these raster layers using the R package *dismo*^90^.

Pearson-Correlation tests were performed between the environmental variables (explanatory variables) and the expression of meiotic and apomictic pathways (response variables) at ovule and seed stages. A generalized linear model (GLM) applied on explanatory variables showing a significant correlation to observations exhibited a nonlinear pattern and thus, a nonlinear regression model was used to determine best-fitting parameters and predict responses of reproductive modes. Parameter values providing the best fit were obtained using a grid search and by minimization of the residual sums of squares (RSS)^91^. The function *nls2* of the R package nls2^92^ was used for the grid search and the nonlinear regression. The function curve in stats R package^93^ was used to add the curve described by the mean function to the plot and to predict expected reproductive mode proportions for different environmental conditions as well as to test the model using observations meiotic and apomictic proportions from common garden experiments. A nonparametric bootstrap analysis of 1000 replicates was performed to test the significance of the gradient value obtained for the mean function.

Analysis of seasonal effects on the incidence of reproductive pathways and reproductive mode efficiency was done by a paired *t*-test and standard Pearson’s Chi-squared test for two time periods (early season: November-December; late season: February-March).

## Supplementary information

Supplementary information.

## Data Availability

All data generated or analyzed during this study are included in this published article [and its supplementary information files]
