# Supplementary material for: Sexual modulation in a polyploid grass: a reproductive contest between environmentally inducible sexual and genetically dominant apomictic pathways
Source: Sci Rep. 2020 May 20;10:8319. doi: 10.1038/s41598-020-64982-6 (PMC7239852; doi:10.1038/s41598-020-64982-6)
Supplement: Supplementary file 1 — Supplementary information. [file 41598_2020_64982_MOESM1_ESM.pdf]

**Supporting Information**

Article title: **Sexual modulation in a polyploid grass: a reproductive contest between environmentally inducible sexual and genetically dominant apomictic pathways**

Authors: Piyal Karunarathne1,2*, Anna V. Reutemann3, Mara Schedler3, Adriana Glücksberg3, Eric J. Martínez3, Ana I. Honfi4, and Diego H. Hojsgaard1

The following Supporting Information is available for this article:

**Figure S1.** Overall trend of the observed reproductive potential x reproductive efficiency values for each reproductive pathway plotted against MDR zones. **a:** apomixis; **b:** sexuality.

**Figure S2.** Plot depicting the nonlinear curve fitting of mean functions explaining the observed response of meiotic and apomictic embryo sacs proportions under different MDR.

**Figure S3.** Response of sexual reproductive efficiency from populations sharing similar sexual reproductive potential located under different MDR zones.

**Table S1.** All the studied populations of *Paspalum intermedium* in the study, including locations, geographic coordinates and voucher codes.

**Table S2.** Proportion of sexual (meiotic) and apomictic reproductive pathways in ovules at blooming (mature embryo sacs) and seed stages of the studied *P. intermedium* populations.

**Table S3.** Reproductive pathways fitness analysis of the studied *P. intermedium* populations

**Table S4.** Pearson correlation of meiotic ($r_{sex}$) and apomictic ($r_{apo}$) proportions at ovule and seed stages to different climatic variables.

**Table S5.** Proportion of sexual (meiotic) and apomictic reproductive pathways in *P. intermedium* populations under different MDR zones.

**Methods S1** Nonlinear function explaining environmental influence on reproductive pathways

**Fig. S1.** Overall trend of the observed reproductive potential x reproductive efficiency values for each reproductive pathway plotted against MDR zones. **a:** apomixis; **b:** sexuality.

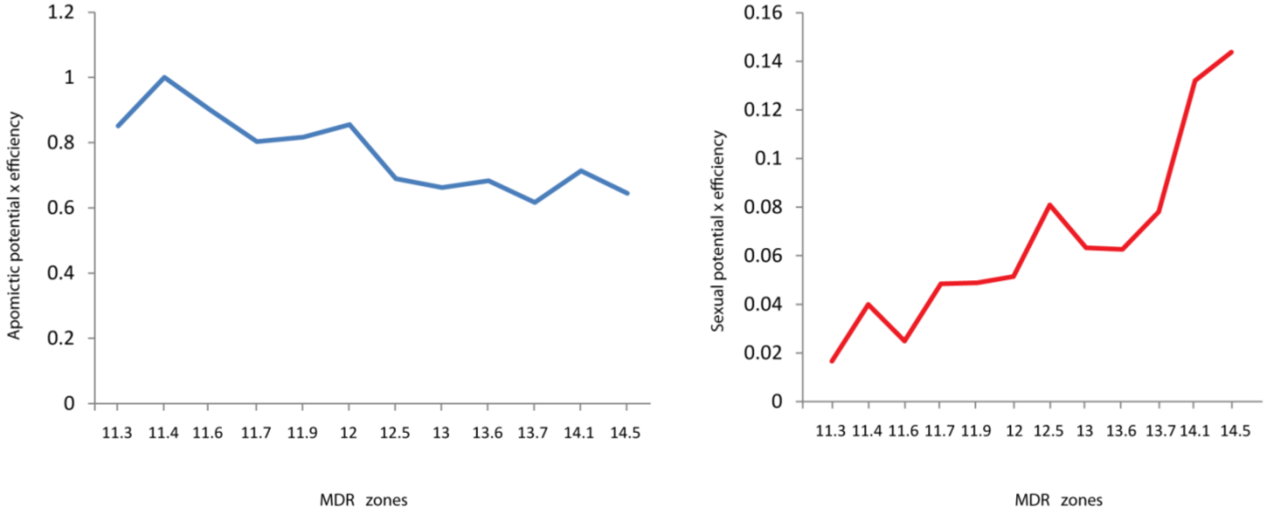


**Fig. S2.** Plot depicting the nonlinear curve fitting of mean functions explaining the observed response of meiotic and apomictic embryo sacs proportions observed under different mean diurnal ranges in the studied area.

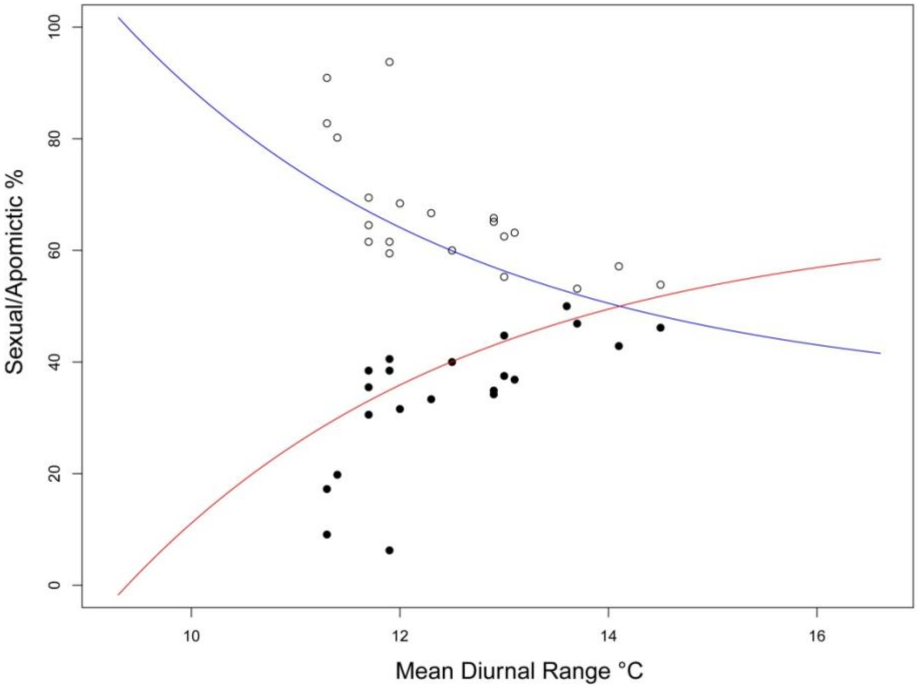

**Fig. S3.** Response of sexual reproductive efficiency from populations sharing similar sexual reproductive potential located under different MDR zones. **a:** three populations between 11.7°C-12.5°C MDR zones and with 0.41±0.01 sexual potential. **b:** four populations between 11.7°C-13.7°C MDR zones and with 0.53±0.01 sexual potential. **c:** three populations between 11.4°C-14.5°C MDR zones and with 0.65±0.02 sexual potential.

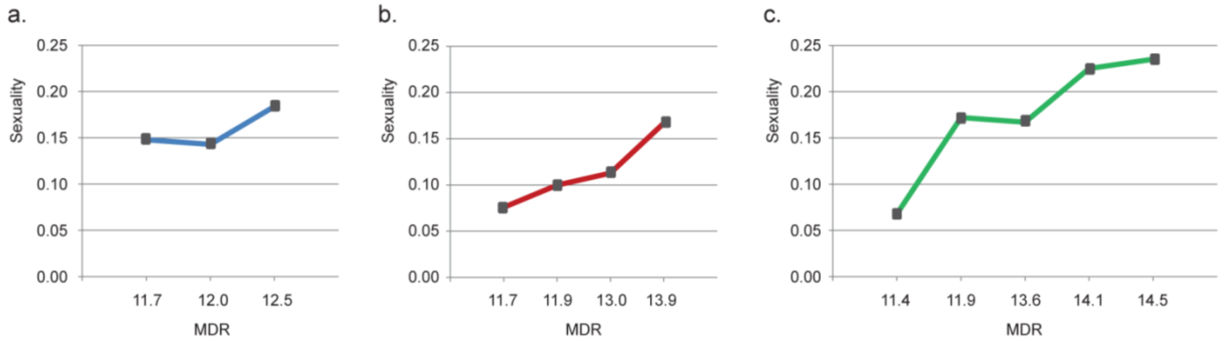


**Table S1** All the studied populations of *Paspalum intermedium* in the study, including locations, geographic coordinates and voucher codes.

| Col._code | Pop. Label | Longitude | Latitude | Location and vouchers | Ploidy (x=10) |
|---|---|---|---|---|---|
| Hojs401 | 1A | -57.0760 | -27.5831 | Ctes, NR12, b/ Ituzaingó and Itá Ibaté HojsHonMar401 (CTES, MNES) | 4x |
| M29 | 1B | -56.8626 | -29.4254 | Ctes, Dpt Gral San Martín, NR14, 4km South-western PR126 intersection MarSched29 (CTES) | 4x |
| Hojs402 | 1C | -57.2814 | -29.6079 | Ctes, PR123, b/access to NR14 Aguapey creek HojsHonMar402 (CTES, MNES) | 4x |
| Hojs403 | 1D | -57.4844 | -29.8267 | Ctes, PR126, b/ Curuzú Cuatiá and Paso de los Libres HojsHonMar403 (CTES, MNES) | 4x |
| Hojs404 | 1E | -58.2093 | -29.9081 | Ctes, PR126, b/ Curuzú Cuatiá and Sauce HojsHonMar404 (CTES, MNES) | 4x |
| Hojs405 | 1F | -58.7083 | -30.1007 | Ctes, Dpt Sauce, PR126, 9 km of Sauce, b/ C.Cuatiá and Sauce HojsHonMar405 (CTES, MNES) | 4x |
| Hojs406 | 1G | -58.8357 | -29.8732 | Ctes, Dpt Sauce, PR23, b/ Barrancas´creek and Perugorría HojsHonMar406 (CTES, MNES) | 4x |

| | | | | | |
|---|---|---|---|---|---|
| Hojs409 | 1H | -58.65584 | -29.4700 | Ctes, Dpt Curuzú Cuatiá, PR23, 16 km from Perugorría<br>HojsHonMar409 (CTES, MNES) | 4x |
| Hojs410 | 1I | -58.65475 | -29.2953 | Ctes, PR24, b/Perugorría intersection to NR12<br>HojsHonMar410 | 4x |
| Hojs414 | 1J | -59.1137 | -27.5268 | Chaco, NR11, near Resistencia<br>HojsSchedMar414 (CTES, MNES) | 4x |
| Hojs415 | 1K | -59.34552 | -28.4824 | Sta Fe, Villa Ocampo, NR11 and access to slaughterhouse<br>HojsSchedMar415 (CTES, MNES) | 4x |
| Hojs416 | 1M | -59.77002 | -29.5801 | Sta Fe, PR1, 3 km Southern Romang (1 km before El Gusano creek)<br>HojsSchedMar416 (CTES, MNES) | 4x |
| Hojs420 | 1P | -59.63136 | -28.5292 | Sta Fe, PR32, 5 Km Southern Villa Ana | 2x |
| Hojs422 | 1Q | -59.61130 | -27.0991 | Chaco, NR16, 73 km North-eastern from Resistencia<br>HojsSchedMar422 (CTES, MNES) | 2x |
| Hojs423 | 1R | -59.73522 | -25.2539 | Formosa, NR95, 13 km Eastern Ibarreta | 2x |
| Hojs424 | 1S | -58.9320 | -24.9565 | Formosa, NR86,41 km North-western Espinillo<br>HojsSchedMar424 (CTES, MNES) | 4x |
| Hojs425 | 1T | -58.1383 | -25.4033 | Formosa, PR6, 15 km Eastern Riacho He He | 2x |
| Hojs429 | 1U | -58.1427 | -25.9639 | Formosa, NR11, 25 km Northern Formosa´s city | 2x |
| Hojs432 | 1V | -58.9334 | -25.7758 | Formosa, NR81, 89 km North-western Formosa´s city | 2x |
| M31 | 1W | -59.2832 | -26.0011 | Formosa, PR3<br>MarSched31 (CTES) | 2x |
| M26 | 1X | -59.3546 | -26.2404 | Formosa, PR3, 9 km Northern El Colorado town<br>MarSched26 (CTES) | 2x |
| Hojs440 | 2C | -59.8931 | -27.7347 | Chaco, PR7, towards La Sabana settlement | 4x |
| Hojs443 | 2F | -60.0928 | -28.7823 | Sta Fe, PR3, Southern La Colmena settlement<br>HojsKaruSchedMar443 (CTES, MNES) | 4x |
| Hojs445 | 2H | -60.1769 | -29.2907 | Sta Fe, PR3, 2,5 Km after Toba asttlement, 21 km Northern Vera<br>HojsKaruSchedMar445 (CTES, MNES) | 4x |

| | | | | | |
|---|---|---|---|---|---|
| Hojs451 | 2M | -61.1900 | -30.5030 | Sta Fe, PR4, 21 km Southern San Cristobar HojsKaruSchedMar451 (CTES, MNES) | 4x |
| Hojs453 | 2Ñ | -61.0815 | -29.3140 | Sta Fe, PR13, 3-5 km before intersect NR98 towards Chaco HojsKaruSchedMar453 (CTES, MNES) | 4x |
| Hojs454 | 2O | -60.9924 | -29.1106 | Sta Fe, PR13, 20 km North of NR98 intersection | 4x |
| Hojs455 | 2P | -60.7479 | -28.1586 | Sta Fe, PR13, 18 km before the border with Chaco HojsKaruSchedMar455 (CTES, MNES) | 4x |
| Hojs456 | 2Q | -60.6998 | -27.4953 | Chaco, NR95, 10 km after Villa Angela, towards R.S. Peña HojsKaruSchedMar456 (CTES, MNES) | 2x, 4x |
| Hojs465 | 2R | -60.1767 | -26.8977 | Chaco, NR16 and intersection to Colonia Aborigen, from R.S. Peña towards Resistencia HojsKaruSchedMar465 (CTES, MNES) | 4x |
| Hojs468 | 2S | -58.7822 | -26.8863 | Chaco, NR11, 66 km Northern Resistencia HojsKaruSchedMar468 (CTES, MNES) | 2x |
| Hojs470 | 2T | -58.1248 | -27.7428 | Ctes, PR5, 53 km from San Luis del Palmar towards Caá Catí HojsKaruHonMar470 (CTES, MNES) | 2x, 3x, 4x |
| Hojs475 | 2U | -58.0026 | -31.2670 | Entre Rios, dirt road on Monseñor Ricardo Rösch Avenue HojsKaruHonMar475 (CTES, MNES) | 4x |
| Hojs478 | 2V | -56.3832 | -28.7934 | Ctes,NR14, 37 km Northern Alvear town HojsKaruHonMar478 (CTES, MNES) | 4x |
| Hojs481 | 2W | -55.9309 | -28.3438 | Ctes, PR94, 27 km Northern Santo Tome´s city, after PR174 intersection HojsKaruMar481 (CTES, MNES) | 2x, 4x |
| Hojs471 | 2X | -57.9346 | -28.3483 | Ctes. Rita pcial 6-1 Concepcion a 9 km | 4x |
| Hojs487 | 2Y | -58.7454 | -28.1656 | Ctes, NR12, 16 km North-western Saladas, milestone km 951 | 2x, 4x |
| M9 | | -58.3854 | -27.6132 | Corrientes, PR5, 21 km from San Luis del Palmar towards Caá Cati | 2x |

Col._Code: collection code; Pop. Label: population label; NR: national route; PR: provincial route; b/: between; Hojs: Hojsgaard D; Sched: Schedler M; Mar: Martínez E; Hon: Honfi A; Karu: Karunarathne P. Acronyms follow the *Index Herbariorum* (Thiers 2017)

**Table S2.** Proportion of sexual (meiotic) and apomictic reproductive pathways in ovules at blooming (mature embryo sacs) and seed stages of the studied *P. intermedium* populations.

| **Collection code (label)** | **ES proportions** | | **Reprod. efficiency** | | **Potential for†** | | **Multiple AES§** |
|---|---|---|---|---|---|---|---|
| | ***Meiotic*** | ***Apomictic*** | ***Sexual*** | ***Apomictic*** | ***Sexuality*** | ***Apomixis*** | |
| Hoj402(1C) | 0.161 | 0.839 | 0.981 | 1.004 | 0.178 | 0.857 | 0.071 |
| Hoj414(1J) | 0.046 | 0.930 | 0.719 | 1.029 | 0.1 | 1 | 0.65 |
| Hoj403(1D) | 0.304 | 0.652 | 0.164 | 1.594 | 0.7 | 1 | 0.2 |
| Hoj470(2T) | 0.654 | 0.308 | 0.491 | 2.072 | 0.85 | 0.4 | 0 |
| Hoj404(1E) | 0.294 | 0.706 | 0.196 | 1.507 | 0.555 | 0.889 | 0.222 |
| Hoj409(1H) | 0.244 | 0.755 | 0.198 | 1.355 | 0.367 | 0.833 | 0.267 |
| Hoj410(1I) | 0.305 | 0.694 | 0.74 | 1.144 | 0.423 | 0.769 | 0.154 |
| Hoj405(1F) | 0.385 | 0.615 | 0.249 | 1.503 | 0.5 | 0.75 | 0 |
| Hoj478(2V) | 0.043 | 0.956 | 0.593 | 1.027 | 0.062 | 0.937 | 0.437 |
| Hoj471(2X) | 0.319 | 0.617 | 0.423 | 1.396 | 0.6 | 0.88 | 0.24 |
| Hoj415(1K) | 0.267 | 0.733 | 0.45 | 1.256 | 0.428 | 0.928 | 0.143 |
| Hoj475(2U) | 0.385 | 0.577 | 0.459 | 1.361 | 0.417 | 0.625 | 0.0417 |
| Hoj424(1S) | 0.349 | 0.651 | 0.342 | 1.397 | 0.5 | 0.833 | 0.1 |
| Hoj445(2H) | 0.383 | 0.596 | 0.215 | 1.636 | 0.567 | 0.7 | 0.067 |
| Hoj465(2R) | 0.465 | 0.535 | 0.152 | 1.848 | 0.667 | 0.667 | 0.033 |
| Hoj453(2Ñ) | 0.375 | 0.575 | 0.355 | 1.569 | 0.517 | 0.586 | 0.207 |
| Hoj455(2P) | 0.328 | 0.641 | 0.525 | 1.358 | 0.7 | 0.933 | 0.2 |
| Hoj456(2Q) | 0.353 | 0.529 | 0.509 | 1.423 | 0.6 | 0.7 | 0.2 |

†: the potential for each reproductive pathway includes those ovules with both MES and AES;
§: values for the proportion of ovules carrying more than one AES among total numbers of ovules analyzed per population;

**Table S3.** Reproductive pathways fitness analysis of the studied *P. intermedium* populations.

| Col._code | Pop. Label | Ploidy (x=10) | No. of seed sets (full)/infl. | full seeds (%) | No. of infl./ind. | Germinability | Relative Fitness |
|---|---|---|---|---|---|---|---|
| Hojs420 | 1P | 2x | 3866.8 | 32.96 | 45.7 | 0.781 | 0.444 |
| Hojs422 | 1Q | 2x | 4872.2 | 37.46 | 47.0 | 0.781 | 0.435 |
| Hojs423 | 1R | 2x | 6047.6 | 25.55 | 43.7 | 0.552 | 0.235 |
| Hojs429 | 1U | 2x | 5335.1 | 24.95 | 14.3 | 0.757 | 0.103 |
| M31 | 1W | 2x | 4703.8 | 46.75 | 32.7 | 0.698 | 0.318 |
| M26 | 1X | 2x | 4934.0 | 29.73 | 59.7 | 0.781 | 0.412 |
| Hojs468 | 2S | 2x | 6653.0 | 21.70 | 11.0 | 0.630 | 0.066 |
| M9 | | 2x | 3339.1 | 40.70 | 83.3 | 0.800 | 0.427 |
| Hojs470 | 2T | 2x, 3x, 4x | 2590.0 | 39.55 | 8.0 | 0.710 | 0.039 |
| Hojs456 | 2Q | 2x, 4x | 3255.3 | 4.10 | 11.5 | 0.550 | 0.006 |
| Hojs481 | 2W | 2x, 4x | 3934.4 | 10.19 | 14.7 | 0.829 | 0.031 |
| Hojs487 | 2Y | 2x, 4x | 5563.8 | 22.33 | 38.3 | 0.807 | 0.388 |
| M29 | 1B | 4x | 3910.6 | 22.86 | 60.7 | 0.657 | 0.267 |
| Hojs402 | 1C | 4x | 1475.0 | 2.85 | 20.0 | 0.757 | 0.004 |
| Hojs403 | 1D | 4x | 4984.0 | 3.03 | 10.0 | 0.880 | 0.009 |
| Hojs404 | 1E | 4x | 4305.8 | 13.07 | 17.0 | 0.828 | 0.051 |
| Hojs405 | 1F | 4x | 4123.3 | 22.35 | 12.7 | 0.952 | 0.074 |
| Hojs406 | 1G | 4x | 7598.0 | 22.49 | 44.0 | 0.930 | 0.499 |
| Hojs409 | 1H | 4x | 7486.0 | 6.51 | 17.0 | 0.680 | 0.039 |
| Hojs410 | 1I | 4x | 2645.0 | 23.63 | 23.0 | 0.860 | 0.086 |
| Hojs414 | 1J | 4x | 3021.0 | 32.03 | 26.3 | 0.829 | 0.135 |
| Hojs416 | 1M | 4x | 3156.7 | 7.03 | 20.7 | 0.762 | 0.063 |
| Hojs424 | 1S | 4x | 4387.5 | 18.69 | 32.1 | 0.740 | 0.128 |
| Hojs440 | 2C | 4x | 7038.0 | 8.38 | 52.7 | 0.790 | 0.129 |
| Hojs443 | 2F | 4x | 7129.5 | 4.81 | 65.0 | 0.800 | 0.126 |
| Hojs453 | 2Ñ | 4x | 1549.5 | 18.63 | 47.5 | 0.910 | 0.120 |
| Hojs465 | 2R | 4x | 3198.0 | 23.96 | 43.3 | 0.757 | 0.160 |
| Hojs475 | 2U | 4x | 3161.9 | 5.53 | 18.0 | 0.848 | 0.019 |
| Hojs471 | 2X | 4x | 4825.6 | 13.26 | 23.7 | 0.657 | 0.059 |

Col._Code: collection code; Pop. Label: population label.

**Table S4.** Pearson correlation of meiotic ($r_{sex}$) and apomictic ($r_{apo}$) proportions at ovule and seed stages to different climatic variables.

| Climatic Variable§ | Embryo Sacs | | | Seeds | | |
|---|---|---|---|---|---|---|
| | ***p*-value** | $r_{apo}$ | $r_{sex}$ | ***p*-value** | $r_{apo}$ | $r_{sex}$ |
| BIO1:Annual Mean Temperature | 0.828 | -0.051 | 0.051 | 0.989 | -0.004 | 0.004 |
| **BIO2 : Mean Diurnal Range†** | **0.000** | **-0.699** | **0.679** | **0.030** | **-0.523** | **0.559** |
| BIO3 : Isothermality (BIO2/BIO7) (* 100) | 0.042 | -0.447 | 0.447 | 0.691 | -0.112 | 0.112 |
| BIO4 : Temperature Seasonality (st. dev. *100) | 0.281 | -0.247 | 0.247 | 0.223 | -0.334 | 0.334 |
| BIO5 : Max Temperature of Warmest Month | 0.023 | -0.306 | 0.306 | 0.083 | -0.462 | 0.462 |
| BIO6 : Min Temperature of Coldest Month | 0.074 | 0.399 | -0.399 | 0.174 | 0.371 | -0.371 |
| **BIO7 : Temperature Annual Range (BIO5-BIO6)** | **0.001** | **-0.686** | **0.686** | 0.054 | -0.379 | 0.379 |
| BIO8 : Mean Temperature of Wettest Quarter | 0.037 | -0.452 | 0.458 | 0.068 | -0.365 | 0.325 |
| BIO9 : Mean Temperature of Driest Quarter | 0.939 | 0.018 | -0.018 | 0.766 | 0.084 | -0.084 |
| BIO10 : Mean Temperature of Warmest Quarter | 0.523 | -0.148 | 0.148 | 0.619 | -0.140 | 0.140 |
| BIO11 : Mean Temperature of Coldest Quarter | 0.939 | 0.018 | -0.018 | 0.766 | 0.084 | -0.084 |
| BIO12 : Annual Precipitation | 0.090 | 0.178 | -0.178 | 0.115 | 0.424 | -0.424 |
| BIO13 : Precipitation of Wettest Month | 0.052 | 0.417 | -0.407 | 0.796 | -0.073 | 0.073 |
| **BIO14 : Precipitation of Driest Month** | 0.061 | 0.386 | -0.386 | **0.048** | **0.518** | **-0.518** |
| BIO15 : Precipitation Seasonality (CV) | 0.004 | -0.605 | 0.605 | 0.051 | -0.332 | 0.332 |
| BIO16 : Precipitation of Wettest Quarter | 0.005 | 0.591 | -0.591 | 0.698 | 0.109 | -0.109 |
| **BIO17 : Precipitation of Driest Quarter** | **0.001** | **0.668** | **-0.668** | 0.077 | 0.370 | -0.370 |
| BIO18 : Precipitation of Warmest Quarter | 0.648 | 0.106 | -0.106 | 0.295 | -0.289 | 0.289 |
| **BIO19 : Precipitation of Coldest Quarter** | **0.001** | **0.668** | **-0.668** | 0.077 | 0.370 | -0.370 |
| UV-B Radiation | 0.388 | -0.199 | 0.199 | 0.567 | -0.161 | 0.161 |
| Elevation | 0.508 | -0.153 | 0.153 | 0.414 | 0.228 | -0.228 |
| Photosynthetically Active Radiation (PAR) | 0.925 | 0.022 | -0.022 | 0.708 | 0.106 | -0.106 |
| Cloud Cover (%) | 0.069 | 0.275 | -0.263 | 0.233 | 0.328 | -0.328 |
| Frost Day Frequency (no. of days) | 0.102 | -0.367 | 0.367 | 0.536 | -0.174 | 0.174 |
| Vapor pressure at ground level | 0.663 | 0.101 | -0.101 | 0.475 | 0.200 | -0.200 |

**§:** Bioclimatic variables are as in WorlClim data base; †: Mean of monthly diurnal temperature range (max temp - min temp);

**Table S5.** Proportion of sexual (meiotic) and apomictic reproductive pathways in *P. intermedium* populations under different MDR zones.

| Col. Code (pop. label) | Condition | Bio2 (MDR) | Sexual | Apomictic |
|---|---|---|---|---|
| Hoj402(1C) | natural | 11.3 | 0.169 | 0.831 |
| Hoj414(1J) | natural | 11.3 | 0.067 | 0.933 |
| Hoj403(1D) | natural | 11.4 | 0.068 | 0.932 |
| Hoj470(2T) | natural | 11.6 | 0.333 | 0.667 |
| Hoj404(1E) | natural | 11.7 | 0.076 | 0.924 |
| Hoj409(1H) | natural | 11.7 | 0.061 | 0.939 |
| Hoj410(1I) | natural | 11.7 | 0.263 | 0.737 |
| Hoj405(1F) | natural | 11.9 | 0.1 | 0.9 |
| Hoj478(2V) | natural | 11.9 | 0.037 | 0.963 |
| Hoj471(2X) | natural | 11.9 | 0.172 | 0.828 |
| Hoj415(1K) | natural | 12 | 0.143 | 0.857 |
| Hoj475(2U) | natural | 12.5 | 0.184 | 0.816 |
| Hoj424(1S) | natural | 13 | 0.129 | 0.871 |
| Hoj445(2H) | natural | 13 | 0.096 | 0.904 |
| Hoj465(2R) | natural | 13.6 | 0.076 | 0.924 |
| Hoj453(2Ñ) | natural | 13.7 | 0.167 | 0.833 |
| Hoj455(2P) | natural | 14.1 | 0.225 | 0.775 |
| Hoj456(2Q) | natural | 14.5 | 0.235 | 0.765 |
| Hoj414(J15) | cultivated | 11.1 | 0.0 | 1.0 |
| Hoj414(J8) | cultivated | 11.1 | 0.077 | 0.923 |
| Hoj445 (2H) | cultivated | 11.1 | 0.0 | 1.0 |
| Hoj404(E4) | cultivated | 11.1 | 0.188 | 0.812 |
| Hoj404(E19) | cultivated | 11.1 | 0.125 | 0.875 |
| Hoj453(2Ñ) | cultivated | 11.1 | 0.15 | 0.85 |

**Methods S1** Nonlinear function explaining environmental influence on reproductive pathways

In order to better understand the ecological influence on reproductive modes and to model its possible responses, a nonlinear function for the observed expression of meiotic and apomictic pathways and the MDR was formulated. The nonlinear equation best explaining our results takes the form of an exponential increase/decay with a horizontal asymptote. For apomixis it can be written as

$$A_{(d)} = A_m + A_0 \bullet e\ (-k^{(d-\ d0)})$$

Where, $A_m$ is the minimum AES%, $A_0$ is the maximum AES%, $k$ is the gradient constant, $d$ is diurnal variation (MDR) and the $d_0$ is the temperature at which AES% is 100%. The values obtained from the grid search (see Materials and Methods) were, $A_m$ = 30%, $k$ = 0.21 and $d_0$ = 7.8; $A_0$ was assumed to be 100 as it is the maximum theoretical AES% possible. Therefore, the equation can be written as

$$A_{(d)} = 30 + 100 \bullet e^{\ (-0.21(d-7.8))} \qquad \text{(see Figure 4)}$$

The same procedure was followed for the seed data. However, all of our attempts to formulate a non-linear model equation failed to converge, presumably due to the lack of data points along the MDR zones. From the similarity of the trend lines observed for the ovule stage between the GLM and non-linear model we can assume a similar non-linear behavior for the seed stage respect to MDR.
